# The HuR CMLD-2 inhibitor exhibits antitumor effects via MAD2 downregulation in thyroid cancer cells

**DOI:** 10.1038/s41598-019-43894-0

**Published:** 2019-05-14

**Authors:** Lorenzo Allegri, Federica Baldan, Sudeshna Roy, Jeffrey Aubé, Diego Russo, Sebastiano Filetti, Giuseppe Damante

**Affiliations:** 1 Department of Medical Area, University of Udine, 33100 Udine, Italy; 2 Department of Translational and Precision Medicine, University of Roma ‘Sapienza’, 06100 Roma, Italy; 3 Department of BioMelecular Sciences, School of Pharmacy, University of Mississippi, 413 Faser Hall, Mississippi, 38677-1848 USA; 4 Division of Chemical Biology and Medical Chemistry, UNC Eshelman School of Pharmacy, University of North Carolina at Chapel Hill, North Carolina, 27599-7363 USA; 5 Department of Health Sciences, University of Catanzaro “Magna Graecia”, 88100 Catanzaro, Italy

**Keywords:** Targeted therapies, Thyroid cancer

## Abstract

Hu antigen R (HuR) is indeed one of the most studied RNA-binding protein (RBP) since its fundamental role both in tumorigenesis and cancer progression. For this reason, downregulation in HuR protein levels or inhibition of HuR biological function are, nowadays, attractive goals in cancer research. Here, we examined the antitumor effects of CMLD-2 in four thyroid cancer cell lines (SW1736, 8505 C, BCPAP and K1). Indeed, CMLD-2 competitively binds HuR protein disrupting its interaction with RNA-targets. 35 μM CLMD-2 produced a significant downregulation in thyroid cancer cell viability, coupled to an increase in apoptosis. Moreover, CMLD-2 treatment hindered both migration and colony formation ability. MAD2 is a microtubules-associated protein known to be greatly overexpressed in cancer and correlating with tumor aggressiveness. Furthermore, MAD2 is known to be a HuR target. CMLD-2 treatment induced a strong MAD2 downregulation and rescue experiments depicted it as a key effector in HuR-mediated in cancer. Altogether, these data contributed to foster HuR inhibition as valid antineoplastic treatment in thyroid cancer, highlighting MAD2 as a novel therapeutic target.

## Introduction

Thyroid cancer is the most frequent endocrine malignancy and its incidence has been enhanced in the last decade^1^. Most of thyroid carcinomas derive from follicular cells and are classified in papillary (PTC), follicular (FTC) and undifferentiated or ATC^2^. This latter constitutes one of the most aggressive and lethal human solid tumor, with a median survival of 5 months and less than 20% of patients survives 12 months^3^. Although most of PTC and FTC, named also differentiated thyroid cancer (DTCs), have a favorable outcome, some of them show an aggressive behavior^4^. Nowadays, the treatment of thyroid cancer involves surgery and radioiodine administration and is effective only for DTCs which are able to concentrate the radioiodine^5^. Nevertheless, these therapeutic approaches are not effective for poor differentiated PTC and ATC^2,3^. Therefore, for the aggressive thyroid cancers refractory to the current treatments, innovative approaches and discovery of novel targets are needed.

One of the most studied RNA-binding protein (RBP) is the Hu antigen R (HuR), a member of the Hu family involved in regulation of several RNA properties, including stability, localization and translation and also involved in tumorigenesis^6,7^. HuR binds to its targets thought two RNA recognition motifs (RRM**)**, RRM1 and RRM2, in correspondence to adenine-uridine rich elements (ARE)^8,9^. HuR binding to ARE-containing mRNAs is generally accepted as leading to mRNA stabilization and increased translation^10,11^. HuR is located into the nucleus and, in response to stimuli, shuttles to the cytoplasm where its targets can be processed^7,12^. Numerous studies demonstrated HuR overexpression and cytoplasmic delocalization in several cancers, including breast cancer, lung adenocarcinoma, ovarian cancer, laryngeal squamous cell cancer and colon cancer^7,12^, which are often associated with cancer progression and worst prognosis^13–16^. In two our previous studies, we demonstrated HuR overexpression was also demonstrated in thyroid cancer^17^ and how its silencing, by RNA interference, induce reduction of cell viability and tumor aggressiveness in different anaplastic thyroid cancer (ATC) cell lines^18^.

Considering the importance of HuR in cancer development and progression, this RBP is considered a promising therapeutic target in cancer treatment, and preclinical studies, by using siRNAs to downregulate HuR, have shown the effectiveness of this approach in various types of cancer, including thyroid^13,18–20^. However, RBPs like HuR are considered “undruggable targets” due to the absence of a binding pocket for target mRNAs. Indeed, there are only few molecules that block HuR interaction with its targets^21–23^. For example, one of most studied HuR inhibitor is MS-444, that prevents HuR homodimerization and disrupts indirectly the HuR–ARE interaction^21^. Recently, a recently reported HuR inhibitor, a cumarin-derived small molecule named CMLD-2, has shown to competitively bind HuR and directly disrupt its target interaction^19^. CMLD-2 exhibited antitumor activity in different cancer cells as colon, pancreatic and lung cancer cell lines, displaying only a reduced cytotoxicity towards normal cells^19,24^. These biological effects of CMLD-2 could be due to a reduced stability of HuR mRNA targets involved in proliferative and anti-apoptotic pathways^19^. Currently, there are no data on effects of CMLD-2 on thyroid cancer cells. For this reason, in this study we investigated the effects of CMLD-2 HuR inhibition on the growth and migration/invasion capacity of several thyroid cancer cell lines, analyzing both biological and molecular mechanism underlying the effects induced by CMLD-2 treatment.

## Results

### Effects of CMLD-2 on cell viability and apoptosis

**Figure 1:**
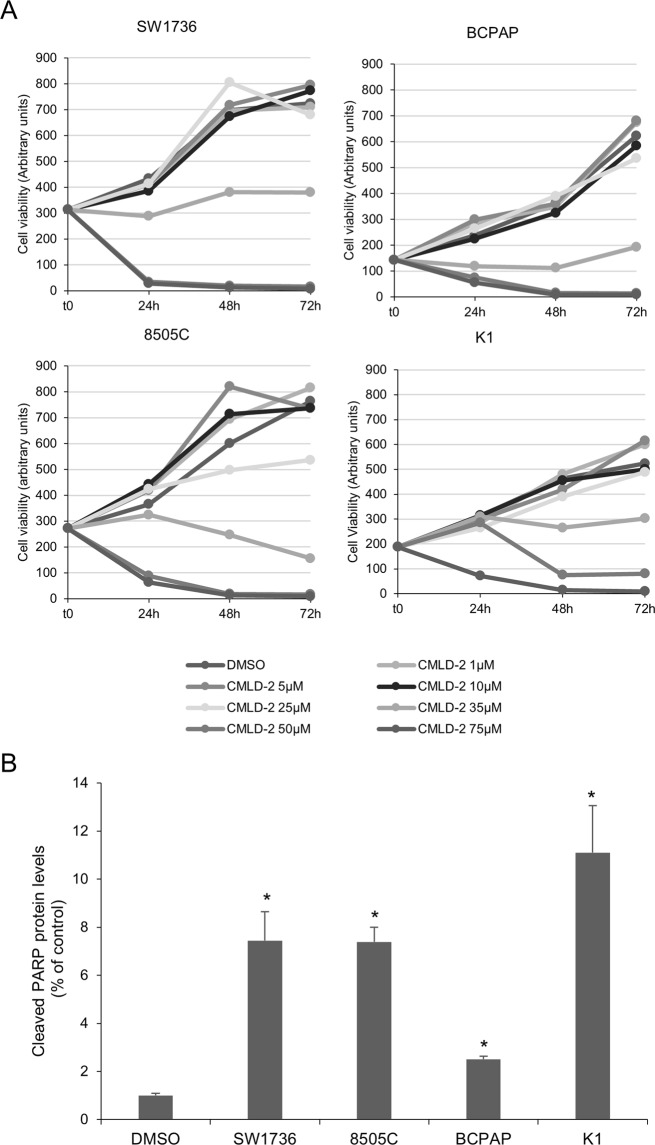
CMLD-2 effects on thyroid cancer cell viability and apoptosis. Panel A. SW1736, 8505 C, BCPAP and K1 cells were treated with CMLD-2 or vehicle (DMSO) at different doses (rising from 1 μM to 75 μM) and at different time (24, 48 or 72 h) and cell viability was analyzed by MTT assay as described in Materials and methods section. Each point represents the mean value of sixfold determinations. SD value was below 10% for all points. Panel B. Densitometric analysis of cleaved PARP fraction levels obtained with Western Blot assay in thyroid cells treated with 35 μM CMLD-2 or vehicle for 72 h. Cleaved PARP protein levels were evaluated as apoptosis marker by Western Blotting, as described in Materials and methods section. For each cell line, the results were normalized against beta-Actin and expressed as percentage over control. P < 0.05, **P < 0.001, ***P < 0.0001 by Student’s t-test.

In a first set of experiments, we evaluated the response to CMLD-2 of several human thyroid cancer cell lines: two derived from ATC (8505 C and SW1736) and two derived from PTC (BCPAP and K1). Initially, we assessed the effects on cell viability of several doses of CMLD-2 in a time course. As shown in Fig. 1 (Fig. 1, panel A), CMLD-2 treatment significantly reduced the viability of all the four cell lines when used at 35, 50 and 75 μM concentration and at different time points. Relying on data obtained, we decided to use for further experiments the median effective dose of 35 μM, i.e. the dose required to achieve 50% of the response in 50% of the four cell line populations. This CMLD-2 concentration is comparable with doses used in other cancer cell lines^19,24^. To evaluate whether the cell viability decrease observed after the treatments was due to apoptotic cell death, a Western blot analysis of cleaved-PARP protein levels was performed (Fig. 1, panel B). In particular, in K1 cells, CMLD-2 treatment induces the most relevant effect with a 11-times cleaved-PARP increment. CMLD-2 shows strong effects also in ATC cell lines (SW1736 and 8505 C) with a 7-times cleaved-PARP increment, while in BCPAP cells its effects are lighter, with a 2.5-times rise of cleaved-PARP.

### Effects of CMLD-2 on tumor aggressiveness

**Figure 2:**
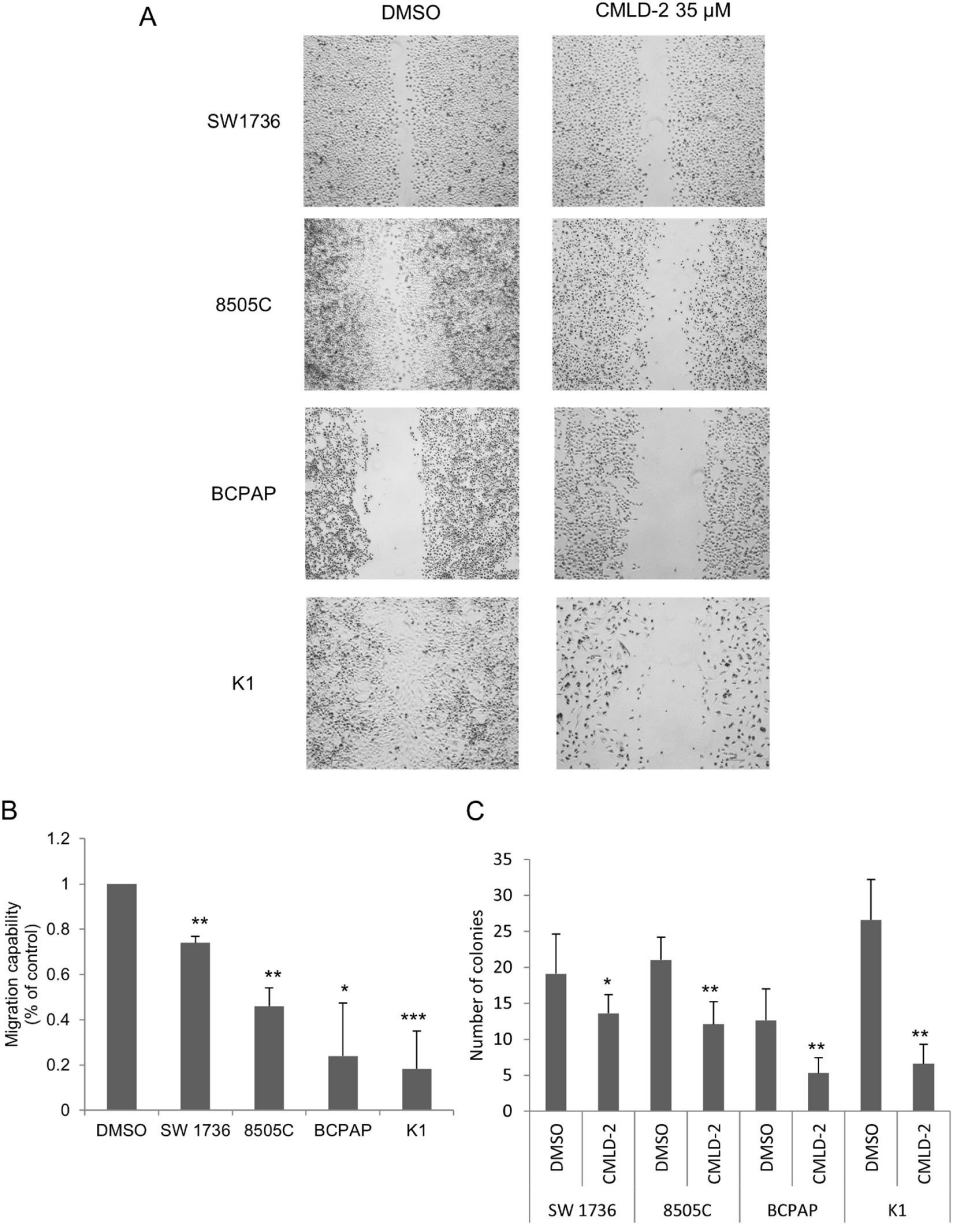
CMLD-2 effects on thyroid cancer cells aggressiveness. SW1736, 8505 C, BCPAP and K1 cells were treated with 35 μM CMLD-2 or DMSO for 72 hours. Panel A. Tumor aggressiveness were evaluated as migration capability by scratch assay. The scratch assay was performed after 72 h of treatment as described in the Material and methods section. After 24 h CMLD-2 treated cells do not fill in the scratch, whereas DMSO treated cells do. Panel B. The histograms represent the migration capability of each cell lines treated either with CMLD-2 or DMSO after 24 h after the scratch. Data are expressed as percentage over control. Panel C. Tumor aggressiveness were evaluated as the clonogenic ability of ATC cells, by soft agar assay. The histogram represents the number of colonies per cell line after the treatments. *P < 0.05, **P < 0.001, ***P < 0.0001 by Student’s t-test. All data are representative of three independent experiments.

To assess CMLD-2 effects on thyroid cancer cells capability to migrate, we performed a scratch assay, a simple method to study directional cell migration *in vitro*. The wound healing ability was evaluated after 35 μl CMLD-2 or vehicle treatment for 72 hours. After scratch creation, cells were monitored for 24 hours and the wound healing was evaluated using an inverted microscope. As shown in Fig. 2 (panels A and B), CMLD-2 35 µM lead a significant decrease of directional migration capability in all the four cell lines. In particular, the migration capability reduction was about 20% in SW1736 cell line, more than 50% in 8505 C and BCPAP and more than 80% in K1 cells.

Then, to better evaluate CMLD-2 effect on tumor cell aggressiveness behavior, we investigated the anchorage-independent growth ability of SW1736, 8505 C, BCPAP and K1 after treatment. For this purpose, we performed a soft agar colony formation assay, a well-established method for characterizing the anchorage-independent growth ability *in vitro*^25^. As shown in Fig. 2 (panel C), after72 h treatment with 35 µM CMLD-2, we observed a strong significant reduction of the number of colonies in all thyroid cancer cells respect to those treated with vehicle only.

### Molecular effects of CMLD-2

**Figure 3:**
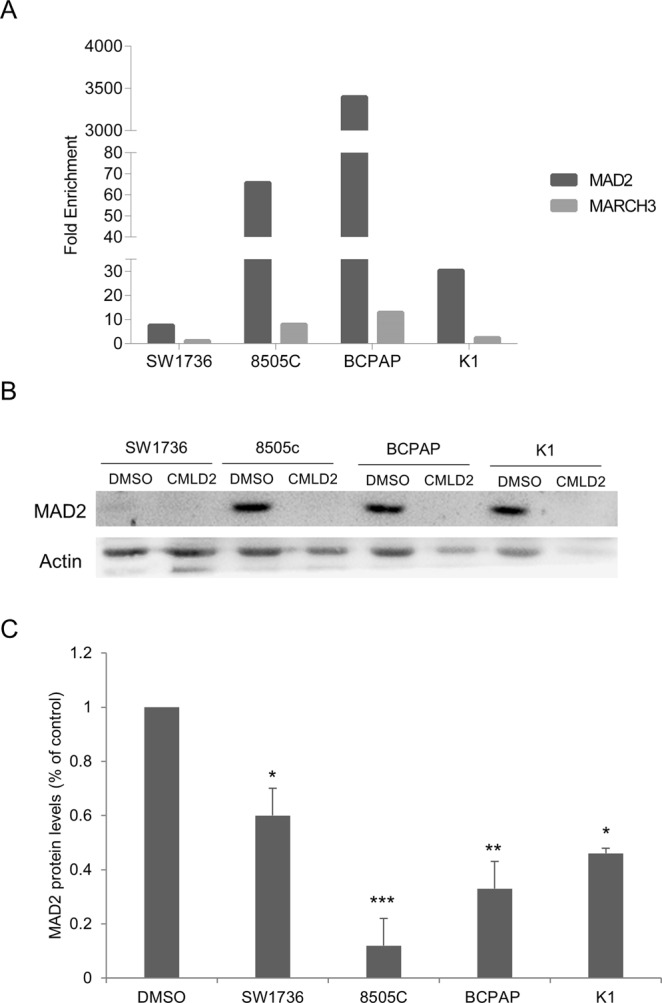
CMLD-2 effects on MAD2 protein levels in thyroid cancer cells. Panel A. MAD2 is identified as HuR target by RIP assay. Thyroid cancer cells were lysed and immunoprecipitated with anti-HuR or anti-IgG antibodies. HuR–bound mRNAs were amplified using specific MAD2 and MARCH3 (negative control) primers and analyzed by qPCR. All samples were run in triplicate. IgG-immunoprecipitate was arbitrarily set at 1.0 and the enrichment was expressed as relative expression value (Fold Enrichment). Panel B. SW1736, 8505 C, BCPAP and K1 cells were treated with DMSO or CMLD-2 35 µM for 72 h. Cells were collected and MAD2 protein levels were analyzed by Western Blot. Panel C. Densitometric analysis of MAD2 protein levels obtained with Western Blot assay. For each cell line, the results were normalized against Actin and expressed as percentage over control. Results are shown as mean ± SD. ***p < 0.001, ****p < 0.0001 by Student’s t-test.

To better understand molecular mechanism underlying CMLD-2 effects on thyroid cancer cells, we focused on MAD2, a HuR-mRNA target known to be involved in cancer^26–28^ and overexpressed in thyroid neoplasms^29^. MAD2 is a key component of the MAD/BUB complex that supervise the sister-chromatid separation during metaphase to anaphase progression^30–32^. First, by a HuR-RIP approach, we immunoprecipitated HuR-bound mRNA and, evaluating MAD2 fold enrichment compared to IgG by qPCR, we confirmed MAD2 as HuR-target (Fig. 3, panel A). Then, we evaluated CMLD-2 effects on MAD2 mRNA and protein levels (Fig. 3, panel B and C). After 48 hours of treatment, CMLD-2 35 µM induces a strong decrease of MAD2 mRNA levels in all the cell lines and in particular in K1, BCPAP and 8505 C cells. These effects reflect those on MAD2 protein levels, that are significantly reduced in all the cell lines, particularly in 8505 C and BCPAP. Relying on data obtained, we focused on the two cell lines showing the highest MAD2 reduction after CMLD2 treatment: 8505 C and BCPAP.

**Figure 4:**
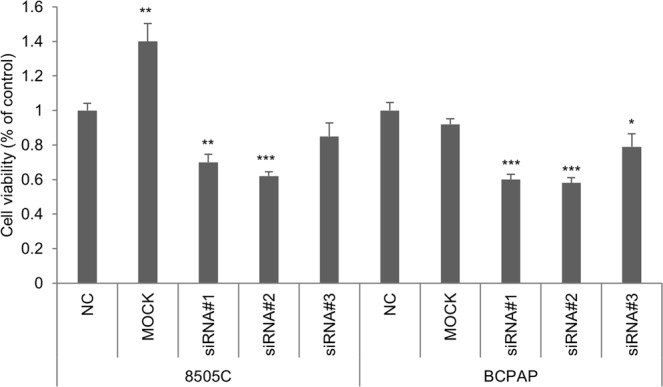
Biological effects of MAD2 silencing in thyroid cancer cells. 8505 C and BCPAP cells were transfected with vehicle (mock), non-targeting siRNA (NC, negative control) or three different siRNA (#1, #2 and #3) sequence specific to MAD2 (5 nM) for 72 h and cell viability was analyzed by MTT assay. All experiments were run in sixfold. P < 0.05, **P < 0.001, ***P < 0.0001 by Student’s t-test.

To evaluate whether the biological effects observed after CMLD-2 administration were, at least in part, due to MAD2 downregulation, we performed an MTT assay after MAD2 silencing by RNA interference. MAD2 was silenced using three different specific siRNA (5 nM) which, as shown in Fig. 4, induce a strong MAD2 reduction in both 8505 C and BCPAP cell lines. We then evaluated cell viability levels after MAD2 silencing and we observed about 40% cell viability decrease in both cell lines after transfection with siRNA#1 and siRNA#2, whereas siRNA#3 seemed to have lower effects.

**Figure 5:**
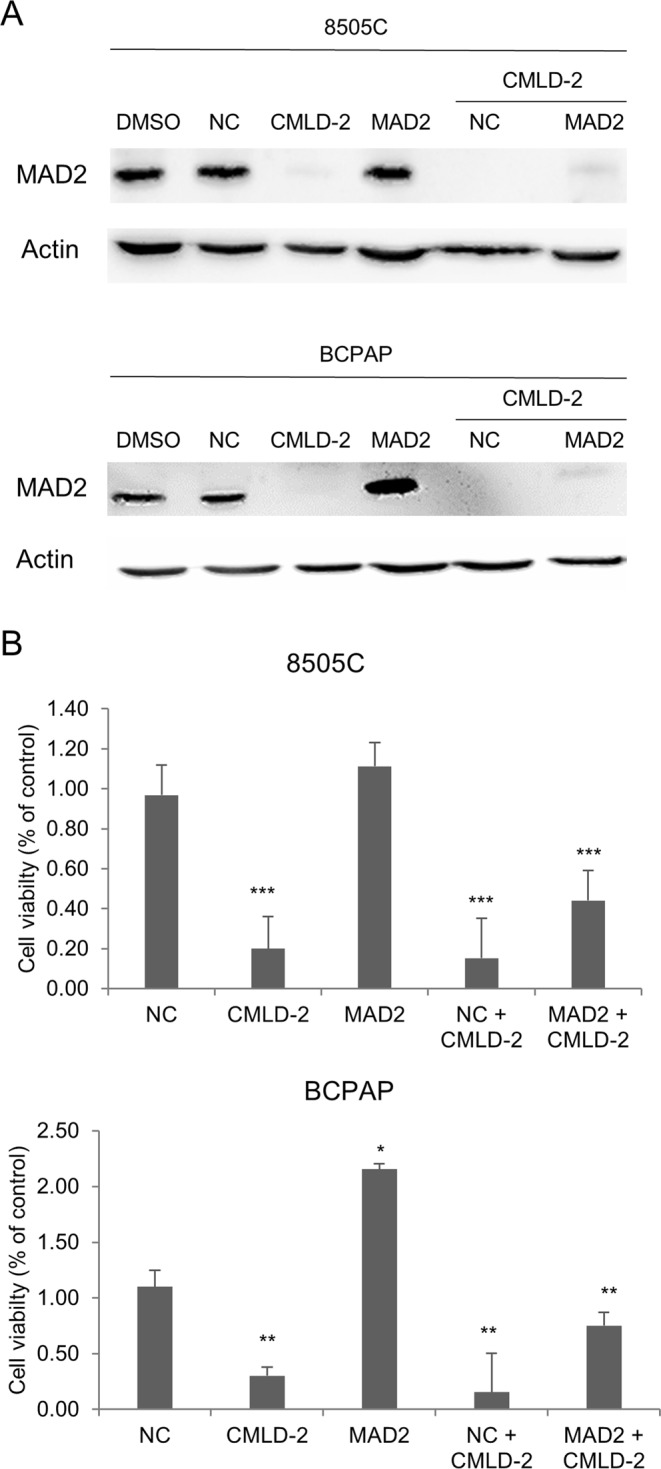
MAD2 is involved in CMLD-2 effects in thyroid cancer cell lines. Panel A. 8505 C and BCPAP cells were treated with DMSO, CMLD-2, pCMV empty vector (NC, negative control), pCMV vector specific for MAD2 (1.3 µg), alone or in combinations. Cells were collected after 72 h treatment and MAD2 protein levels were analyzed by Western Blot. Panel B. 8505 C and BCPAP cells were treated with DMSO, CMLD-2, pCMV empty vector (NC, negative control), pCMV vector specific for MAD2 (1.3 µg), alone or in combinations and cell viability was determined by MTT assay after 72 h. NC was arbitrarily set at 1 and cell viability was expressed as relative expression value. All samples were run in sixfold. P < 0.05, **P < 0.001, ***P < 0.0001 by Student’s t-test.

To confirm the hypothesis that MAD2 would directly be involved in CMLD-2 effects, we performed a rescue experiment. 8505 C and BCPAP cells were treated with pCMV6-MAD2 or pCMV6-empty (NC) and exposed to CMLD-2 for 72 h. As shown in Fig. 5, MAD2 gene over-expression partially rescued its protein levels in CMLD-2-treated cells. To validate that this phenomenon is associated to a cell viability rescue, an MTT assay was performed. 8505 C and BCPAP cells over-expressing MAD2 displayed a significant decrease of CMLD-2 effects when assessing cell viability, about 30% in both 8505 C and BCPAP cells (Fig. 5, panel B).

## Discussion

The search for novel therapeutic approaches for the more aggressive thyroid tumors refractory to the current treatment is an actual challenge and identification of novel molecular targets is an urgent priority in the field of translational research^4,33^. Our previous preclinical studies have shown that HuR is over-expressed in thyroid cancer, suggesting this RNA-binding protein as a novel promising target in thyroid cancer therapy^17^. Several reports have described a pivotal role of HuR in cancer cells and tumors, where its functionally activation represents a pro-survival network for transformed cells^15,34,35^. HuR relevance as prognostic marker has been well documented for several tumor types, included ovarian, lung, colorectal, liver, brain, gallbladder and pancreatic cancer^36–38^. Moreover, several studies have shown that HuR de-regulation in cancer correlates with poor prognosis and therapy resistance^39,40^.

In a recent study, Allegri *et al*. have demonstrated that HuR silencing leads to a decrease in cell viability and tumor aggressiveness and induces apoptosis in thyroid cancer^18^. However, the use of siRNA-mediated HuR-inhibition therapy is still a hard challenge due to the poor cell uptake, low serum stability or difficulty to deliver HuR-siRNA to tumor mass^24^. For these reasons, a pharmacologic inhibition of this RNA-binding protein could be a preferable approach in innovative thyroid cancer therapy. Thus, in this study we focused on CMLD-2, a recently reported compound that competitively binds to HuR and disrupts its target interaction^19^.

In the project initial phase, we demonstrated that CMLD-2 treatment was able to reproduce the effects of HuR silencing in both decreasing the cell viability, by inducing apoptotic processes, and reducing some features of tumor cell aggressiveness. In fact, CMLD-2 treatment significantly reduced colony forming ability and migration capability of thyroid cancer cells. The observed slight differences in the behavior of the cell lines may be due to presence of different genetic alterations, especially in those derived from ATC, although all four cell lines carry the same V600E BRAF mutation, as well as mutation in the TERT gene promoter, known to act as ‘drivers’ of oncogenic transformation in thyroid cancer^41,42^. Anyway, in all cell lines significant changes were observed in both analysis of cell proliferation and migration properties after treatment with treatment with CMLD-2.

In a second experimental setting, we have taken a closer look at the molecular mechanism whereby CMLD-2 causes biological effects on thyroid cancer cells. Our attention was focused on mRNA targets of HuR silencing; analyzing the data of Lebedeva and al., we noticed not only that MAD2 mRNA is an HuR target, but also that both its mRNA and protein levels are diminished after HuR silencing, proving HuR is indispensable for MAD2 expression^43^. MAD2 is the key component of the MAD/BUB complex and one of the key Spindle Assembly Checkpoint (SAC) proteins, responsible for sister-chromatid separation during metaphase to anaphase progression^30–32,44^. The association between MAD2 expression and survival has been examined in several different carcinomas, including colorectal, bladder, testicular, breast and ovarian, where both overexpression and low levels of MAD2 protein have been associated with survival, depending on the tumor type^45^. Wada *et al*. demonstrated that MAD2 is significantly overexpressed in anaplastic thyroid carcinomas than in the differentiated ones (DTCs); furthermore, they showed that MAD2 expressions is higher in advanced DTCs than in non-advanced DTC. These data suggest that MAD2 might be related to advanced thyroid carcinomas, because its overexpression in carcinoma with an aggressive nature^29^. Based on these findings, we first identified MAD2 as a HuR target in all four thyroid cancer cell lines analyzed, by RIP-PCR. Then, we demonstrated that CMLD-2 treatment leads to a strong decrease of MAD2 protein level in thyroid cancer cells.

Since as far as we know there were no data on MAD2 depletion in thyroid cancer cells, we demonstrated the importance of this protein for cell viability, by siRNA silencing and subsequent rescue of MAD2 in our models. MAD2 silenced cells showed a reduced viability, suggesting this protein as one of the most important effectors of CMLD-2-induced cell growth decrease. We further proved that MAD2 is directly involved in the cell viability decrease after CMLD-2 treatment performing a rescue experiment. Thyroid cancer cells over-expressing MAD2 displayed a significant reduction (about 25%) of CMLD-2 effects in terms of cell viability. MAD2 downregulation cannot fully explain the remaining part of CMLD-2 effects, however this is not surprising, since HuR stabilizes a large number of mRNA involved in cell viability, survival and apoptosis. Recent studies have highlighted the link between HuR and the chemokine IL-8 in thyroid cancer^46^. Moreover, Li *et al*. identified IL-8 as HuR-target in pancreatic ductal adenocarcinoma (PDAC) cells^47^. However, in a previous work^17^, we have demonstrated that IL-8 is not a HuR-target in thyroid cells, and this finding is in agreement with those published by Lebedeva *et al*.^43^.

Finally, in this study, we demonstrate, for the first time, the antitumor effects of CMLD-2 in thyroid cancer cell lines, demonstrating that a key molecular mechanism of CMLD-2 effects involved MAD2 down-regulation. When confirmed also *in vivo*, pharmacological HuR inhibition by CMLD-2 could be considered an innovative and promising approach for thyroid cancer treatment.

## Material and Methods

### Cell lines

In this study, we used 4 different thyroid cancer cell lines: SW1736 and 8505 C cell lines, derived from anaplastic thyroid cancer; BCPAP and K1 cell lines, derived from papillary thyroid cancer. All four cell lines carry the same V600E BRAF mutation, and mutation in the TERT gene promoter^41,42^. All cell lines have been validated by short tandem repeat and tested for being mycoplasma-free. K1 cells were grown in DMEM medium (EuroClone, Milan, Italy) while the others were grown in RPMI 1640 medium (EuroClone) Supplemented with 10% fetal bovine serum (Gibco Invitrogen, Milan, Italy), 2 mM L-glutamine (EuroClone) and 50 mg/ml gentamicin (Gibco Invitrogen). Cultured cells were treated either with vehicle (DMSO, Sigma Aldrich, Saint Louis, MO, USA) or CMLD-2, prepared as previously reported^19^. Cells were grown in a humidified incubator (5% CO2 in air at 37 °C) (Eppendorf AG, Hamburg, Germany).

### Cell viability

In order to test cell viability, we applied the Methylthiazolyldiphenyl-tetrazolium bromide (MTT) assay. SW1736, 8505 C, BCPAP and K1 cells (3000 cells/well) were plated onto 96-well plates in 200 μl medium/well and were allowed to attach to the plate for 24 h (t0). Plates were then treated either with vehicle (DMSO) or CMLD-2 at different concentration (rising from 1 μM to 75 μM) and incubated for 24, 48 and 72 hours. 0.5 mg/ml MTT (Sigma-Aldrich) was then added to the cell medium and cells were cultivated for another 4 hours darkened in the cell incubator. The supernatant was removed, 100 μl/well of DMSO (Sigma-Aldrich) were added and the absorbance at 570 nm was measured. All experiments were run in sixfold and cell viability was expressed as a fold change compared to control.

### Protein extraction and Western blot

Thyroid cancer cells were treated with DMSO or 35 μM CMLD-2 for 72 hours. Total protein extraction has been performed, as described previously^48^. Briefly, SW1736, 8505 C, BCPAP and K1 cells by scraping and lysing cells with total lysis buffer (Tris HCl 50 mM pH8, NaCl 120 mM, EDTA 5 mM, Triton 1%, NP40 1%, DTT 1 mM) Supplemented with phenyl-methylsulphonyl fluoride and protease inhibitors. Lysates were then centrifuged at 13000 g for 10 min at 4 °C and supernatants were quantified by Bradford assay.

For Western Blot analysis, proteins were electrophoresed on SDS-PAGE and then transferred to nitrocellulose membranes (GE Healthcare, Little Chalfont, UK), saturated with 5% non-fat dry milk in PBS/0.1% Tween 20. The membranes were then incubated overnight with rabbit polyclonal anti-cleaved-PARP antibody (Abcam, Cambridge, United Kingdom), rabbit anti-actin antibody (Merck KGaA), mouse anti-MAD2 antibody (Santa Cruz Biotechnology, INC). The day after, membranes were incubated with anti-rabbit or anti-mouse immunoglobulin coupled to peroxidase (Merck KGaA) for 2 h. Blots were developed using UVITEC Alliance LD (UVITec Limited, Cambridge, UK) with the SuperSignal Technology (Thermo Scientific Inc Waltham, MA, USA).

### Scratch assay

Thyroid cancer cells migratory ability was evaluated in a “scratch assay” after 72 hours of 35 μM CMLD-2 treatment. Briefly, SW1736, 8505 C, BCPAP and K1 were seeded onto 6-well plates. On the next day, cells were treated with vehicle (DMSO) or CMLD-2 for 72 hours. Then a linear scratch was performed with a 200-μl sterile pipette tip across the cell monolayer. Cells were then incubated in a humidified incubator at 37 °C and images of the scratched monolayer were acquired after 0, 5, 10 and 24 hours with an inverted microscope Leica DMI-600B (Leica Microsystems Ltd.). Experiments were run in triplicate. Differences in filling the scratch were analyzed with ImageJ image analysis software to establish the thyroid cancer cells migration capability after treatment compared to control by using the following formula:

migration rate = (Ato − At)/At0, where At0 represents the initial scratch area, At represents the scratch area measured 5, 10 and 24 hours after.

### Soft agar assay

Thyroid cancer cells clonogenic activity after 35 μM CMLD-2 treatment was evaluated by soft agar assay. Briefly, after 72 h CMLD-2 treatment, cells have been collected and 10000 cells/plate were resuspended in 4 ml of complete medium containing 0.25% agarose and then seeded to the top of a 1% agarose complete medium layer in 6 cm plates. The colonies were counted by the inverted microscope Leica DMI-600B (Leica Microsystems Ltd., Heerbrugg, Switzerland). Data are representative of three independent experiments.

### RNA-binding protein immunoprecipitation

The RNA-binding protein immunoprecipitation (RIP) was performed using EZ-Magna RIP kit (Millipore) according to manufacturer’s instructions, as previously described^17^. Briefly, SW1736, 8505 C, BCPAP and K1 cells were scraped in PBS containing protease inhibitors and then resuspended in RIP lysis buffer (Millipore) containing protease and RNase inhibitors. 5 μg of rabbit polyclonal anti-HuR RIPAb+ antibody (Millipore) or normal Rabbit IgG (Millipore) as negative control were incubated overnight with Magnetic Beads Protein A/G. The day after, samples were added to antibody-bead complexes and incubated overnight. 10% of samples was stored as total input. After washings, immune-complexes and input were eluted and treated with proteinase K and heated at 55 °C for 30 minutes to digest the protein. RNA was purified with phenol/chloroform extraction followed by ethanol precipitation. HuR-bound mRNA were analyzed by gene expression assay and HuR target were identified as fold enrichment compared to IgG.

### Gene expression assays

500 ng of RNA, obtained by RIP, were reverse transcribed to cDNA using random exaprimers and MMLV reverse transcriptase (Life Technologies, Carlsbad, CA, USA). Real-time PCR was performed using Platinum Sybr Green QPCR supermix (Life Technologies) on the ABI Prism 7300 Sequence Detection Systems (Applied Biosystems). MAD2 and MARCH3 oligonucleotide primers were purchased from Sigma-Aldrich and their sequences are available upon request.

### MAD2 silencing

For transient silencing of endogenous MAD2 in 8505c and BCPAP cells, TriFECTa RNAi Kit (Integrated DNA Technologies Inc, Coralville, IA, USA) was used following manufacturer’s instructions. A duplex targeting a site absent in human genome was used as ‘universal’ negative control (NC). Three different siRNA oligonucleotides (siRNA1, siRNA2 and siRNA3) were transfected at 1 nM concentration using DharmaFECT 1 Transfection reagent (Thermo Scientific Inc, Waltham, MA, USA), according to manufacturer’s instructions. The day before transfection, cells were plated in antibiotics-free medium. Cells were harvested 72 h after transfection and gene-silencing efficiency was evaluated by protein levels analysis.

### MAD2 over-expression

To over-express MAD2 protein, we purchased the TrueClone MAD2 cDNA cloned in pCMV6-AC, which contains a full open reading frame of the human Mad2 gene (Origene, Rockville, MD, USA). The over-expression vector was transfected with Turbofect reagent (Fisher Scientific S.A.S., Illkirch, France) at 2 μL per well. Empty-pCMV vector was used as negative control (NC). Once verified the over-expression by Western Blot, we performed a rescue experiment, in order to verify the direct involvement of MAD2 in CMLD-2 HuR inhibition biological effects in thyroid cancer cells. Cells were transfected with pCMV-MAD2 or NC and exposed to 35 μM CMLD2 or vehicle for 72 h. Cell viability was evaluated by MTT assay, as previously described.

### Statistical analysis

All data obtained were expressed as means ± SD, and significances were analyzed with the Student’s t-test performed with GraphPAD Software for Science (San Diego, CA, USA).

## Supplementary information

Supplementary Information
