# Supplementary material for: The HuR CMLD-2 inhibitor exhibits antitumor effects via MAD2 downregulation in thyroid cancer cells
Source: Sci Rep. 2019 May 14;9:7374. doi: 10.1038/s41598-019-43894-0 (PMC6517587; doi:10.1038/s41598-019-43894-0)
Supplement: Supplementary file 1 — Supplementary Information [file 41598_2019_43894_MOESM1_ESM.pdf]

The HuR CMLD-2 inhibitor exhibits antitumor effects via MAD2 downregulation in thyroid cancer cells

Lorenzo Allegri[1]*, Federica Baldan[2]*, Sudeshna Roy[3], Jeffrey Aubé[4], Diego Russo[5], Sebastiano Filetti[2], Giuseppe Damante[1]

[1] Department of Medical Area, University of Udine, 33100 Udine, Italy
[2] Department of Translational and Precision Medicine, University of Roma 'Sapienza', 06100 Roma, Italy
[3] Department of BioMelecular Sciences, School of Pharmacy, University of Mississippi, 413 Faser Hall, Mississippi, 38677-1848, USA
[4] Division of Chemical Biology and Medical Chemistry, UNC Eshelman School of Pharmacy, University of North Carolina at Chapel Hill, North Carolina, 27599-7363, USA
[5] Department of Health Sciences, University of Catanzaro "Magna Graecia", 88100 Catanzaro, Italy;

*Allegri L. and Baldan F. contributed equally to this article.

Corresponding author: Dr. Baldan Federica

Department of Translational and Precision Medicine

Viale del Policlinico 155

00161, Rome - Italy

E-mail: federica.baldan12@gmail.com

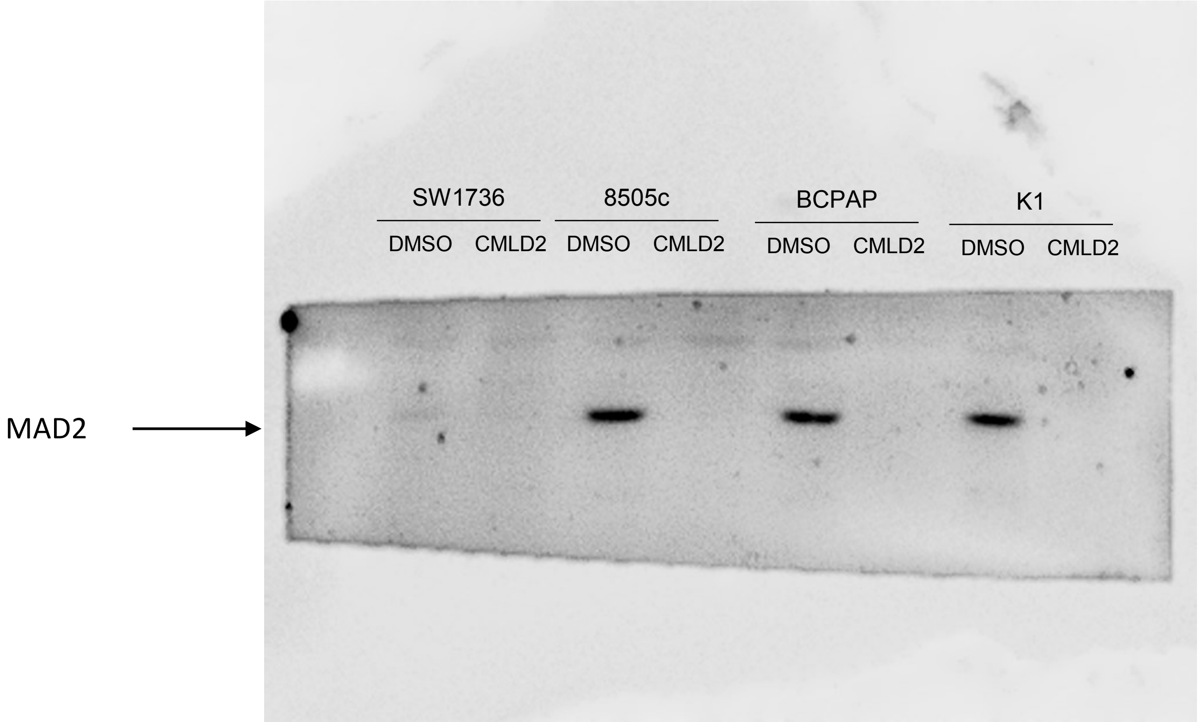

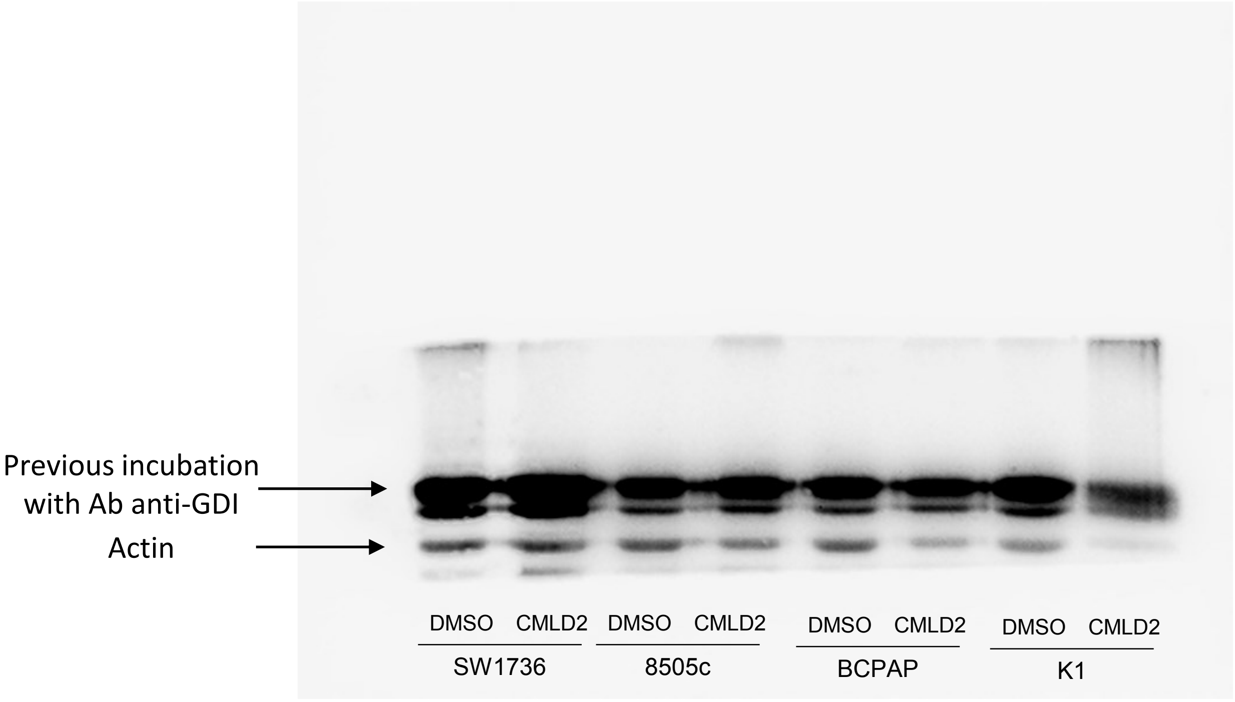

Figure-3 (Baldan)

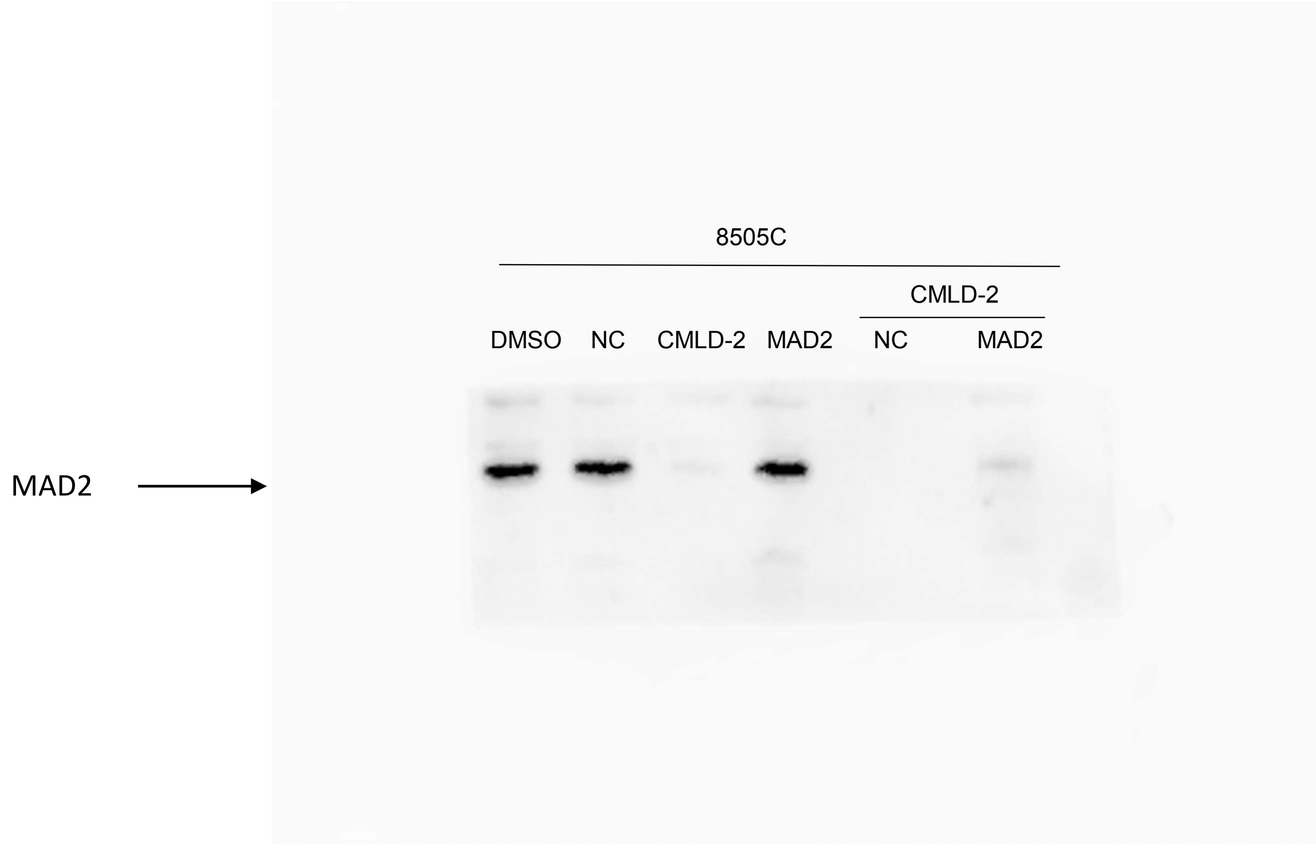

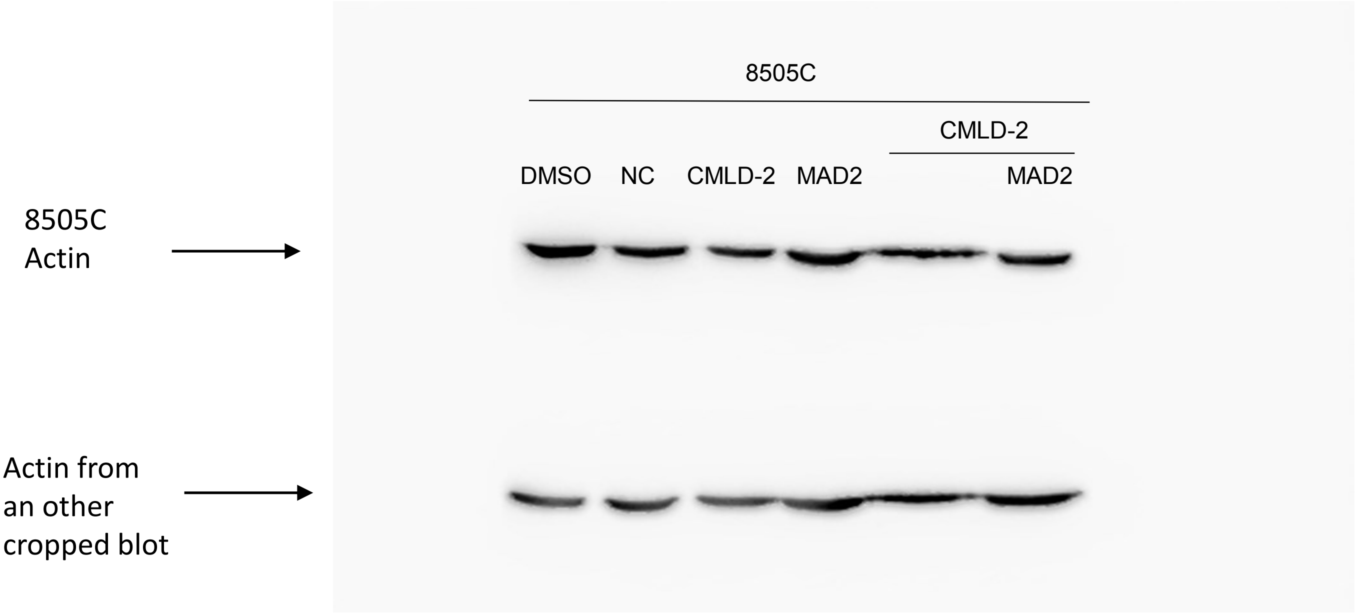

Figure-5 (Baldan)

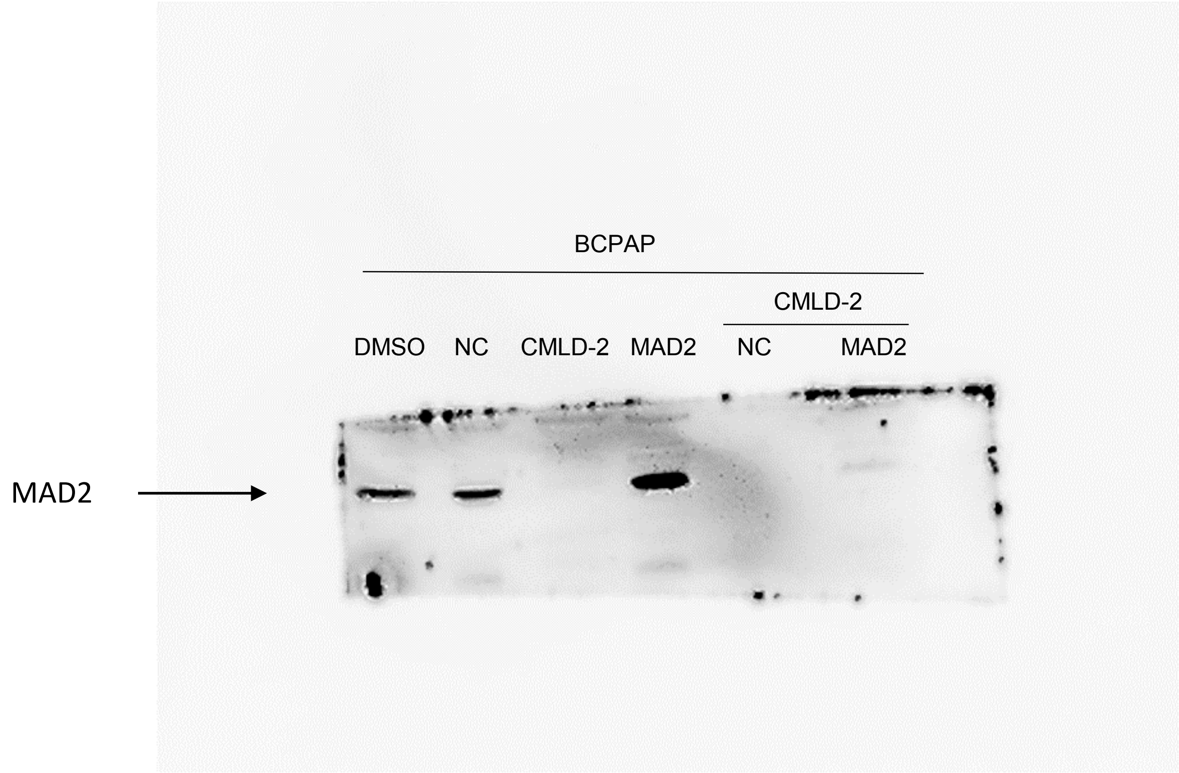

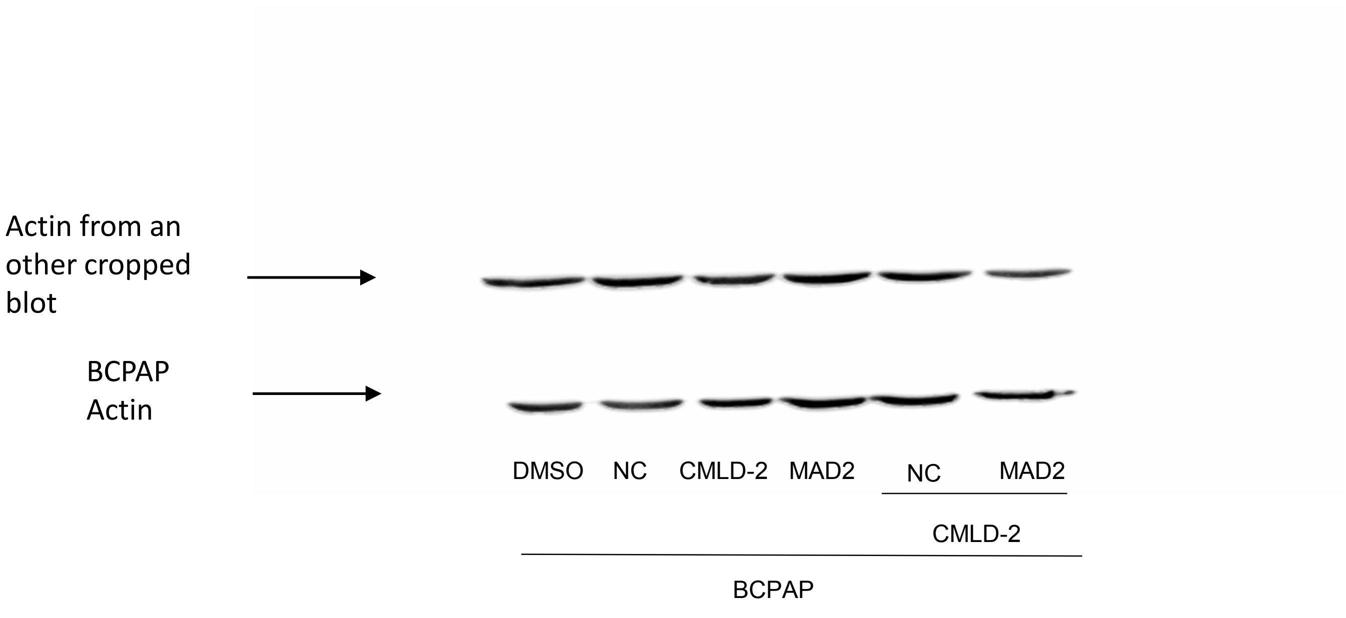

Figure-5 (Baldan)
